# Self-assessment and goal-setting is associated with an improvement in interviewing skills

**DOI:** 10.3402/meo.v19.24407

**Published:** 2014-07-23

**Authors:** Kathleen Hanley, Sondra Zabar, Joseph Charap, Joseph Nicholson, Lindsey Disney, Adina Kalet, Colleen Gillespie

**Affiliations:** Department of Medicine, Division of General Internal Medicine, New York University School of Medicine, New York, NY, USA

**Keywords:** communication skills, self-assessment, peer-assessment, goal setting, videotape review, medical interview

## Abstract

**Purpose:**

Describe the relationship between medical students’ self-assessment and goal-setting (SAGS) skills and development of interviewing skills during the first-year doctoring course.

**Method:**

157 first-year medical students completed three two-case standardized patient (SP) interviews. After each of the first two, students viewed videotapes of their interview, completed a SAGS worksheet, and reviewed a selected tape segment in a seminar. SAGS was categorized into good and poor quality and interviewing skills were rated by trained raters.

**Results:**

SAGS improved over time (37% good week 1 vs. 61% good week 10). Baseline SAGS and interviewing skills were not associated. Initial SAGS quality was associated with change in interviewing skills – those with poor-quality SAGS demonstrated a decrease and those with good-quality SAGS demonstrated an increase in scores by 17 weeks (ANOVA *F=*4.16, p=0.024). For students whose SAGS skills were good at both week 1 and 10, interviewing skills declined in weeks 1–10 and then increased significantly at week 17. For those whose SAGS remained ‘poor’ in weeks 1–10, interviewing skills declined in weeks 10–17.

**Conclusions:**

In general, the quality of students’ SAGS improved over time. Poor baseline SAGS skills and failure to improve were associated with a decrease in interviewing skills at 17 weeks. For students with better SAGS, interviewing skills increased at week 17. Improvement in SAGS skills was not associated with improved interviewing skills. Understanding structured self-assessment skills helps identify student characteristics that influence progressive mastery of communication skills and therefore may inform curriculum and remediation tailoring.

Medical education should ensure that students have life-long, self-directed learning skills essential for continued professional expertise development after formal training has ended (1). The ability to self-monitor and regulate learning through self-assessment and setting goals are key components of achievement in almost all learning domains (2). The development of expertise, as demonstrated through consistent optimal performance of skills, is attained through cycles of challenging practice with accurate feedback from experienced coaches (3). In order to practice safely, it is crucial that physicians develop the capacity and habit to self-monitor their cognitive and non-cognitive expertise. This is particularly true if medical education is to become more flexible and individualized in order to better meet the health needs of society (4).

Effective communication with patients is a critical component of a physician's diagnostic accuracy, therapeutic rapport, and ability to educate and counsel patients (5, 6). Studies suggest that the medical interview is the source of 60–80% of the data needed for diagnosis (7–9). As a result of a hardy research base, there is a fundamental consensus on mastery criteria for interviewing skills and evidence for effective communication curriculum (10–13). Both self-assessment and peer-assessment are commonly incorporated in medical schools’ communication curricula and have been shown to enhance communication skills as measured by performance on Objective Structured Clinical Exams (OSCE) (14). While the evidence is overwhelming that physicians cannot perform *summative* self-assessments accurately, it has been suggested that their situation-relevant self-assessment for the purpose of self-monitoring can be honed and is critical to ongoing learning from experience (15, 16). As Eva and Regehr (17) point out ‘safe practice requires that self-assessment be conceptualized as repeatedly enacted, situationally relevant assessments of self-efficacy and ongoing “reflection-in-practice”’ (17).

There are many reports on the validity and accuracy of medical students self-assessment (1, 18) as well as the contribution of self-assessment programs to ‘promote more mature, collegial and productive learning environments particularly suited to the training of healthcare professionals’ (15).

Chang's cross-sectional study (2011) described content of students’ learning goals and the relationship of students’ learning goals to the students’ interviewing skills in a clinical performance exam (19). However, we found no longitudinal studies such as ours examining the relationship of medical students’ self-assessment and goal-setting (SAGS) ability with the development of clinical skills over time. To refine clinical skills curricula and ensure expertise development, it is important to understand the diversity in and role of students’ capacity for self-assessment, self-monitoring and goal setting in the development of communication skills.

We report on the qualities of students’ goal-setting based on self-assessment of medical interviewing performance and how SAGS are associated with both baseline and subsequent medical interviewing performance in the context of a long established interviewing skills curriculum for first-year medical students.

We introduced a sequence of individual and small-group videotape reviews (VTR) to our first-year doctoring course. The goal of the VTR sessions was to structure students’ self-assessed interviewing skills using literature-based mastery criteria (as reflected in the checklist) and facilitate the development of an effective learning plan. We address three core questions in this study:

1. Does participating in a guided videotape review process of self-assessment and goal setting (SAGS) enhance the quality of students’ SAGS skills?
2. Is the quality of students’ initial SAGS skills associated with concurrent interviewing skills or with changes in interviewing skills over the course of a 17-week, first-year doctoring course?
3. Is improvement in SAGS skills associated with the development of interviewing skills?

## Methods

### Setting and subjects

All 165 first-year medical students at this Northeastern U.S. private medical school are required to successfully complete the 50-h Practice of Medicine (POM) course, which includes modules on doctor–patient communication, physical diagnosis, health policy and preventive medicine, culture and diversity, and ethics. A variety of instructional formats are employed including lectures, small-group seminars (eight students to two faculty), a longitudinal clinical preceptorship, standardized patient (SP) exercises with faculty and SP feedback and small-group videotape review (G-VTR). At the time of this study, a structured self-assessed videotape review (S-VTR) preceding the G-VTR had been recently added.

As part of an ongoing IRB-approved Medical Student Research Registry, all incoming medical students are asked to consent to allow their educational data (routinely collected as part of their medical school experience) to be compiled in a de-identified database and used for research purposes; 157 of 165 students provided this consent and only their data were used in this study.

### Design

This prospective cohort study assessed students’ interviewing skills over three points of time in their first-year doctoring course (initial, Week 10 and Week 17) and assessed their SAGS skills at the first two time points (initial and Week 10).

### Standardized patient exercises

In 2009, POM student class of 2013 participated in three SP case exercises scheduled over a four and a half-month period. The exercises took place over a 2-h period in which each student interacted with each of two SPs for 10 min and then received 4 min of immediate verbal feedback from a faculty observer. In each exercise, at least one of the two SP interactions was faculty observed and one was videotaped. Student participation was required.

Case content and interviewing skills aligned with material covered in the curriculum and were designed to be progressively more challenging over time. SP case training materials were written by course faculty and included a detailed role description. Students were told, in general, what case content to prepare for and in some cases were given brief primers to help them prepare. A previously validated interviewing skills checklist was used consistently across all SP interactions to assess student skills (20–22). SPs participated in both case portrayal training (3 h) and checklist rater training (2 h), both with experienced SP trainers.

### Self-assessment videotape review

Students were assigned the task of reviewing the videotape of their interview on their own time the week following each exercise in preparation for the faculty facilitated Group VTR. Students were given a worksheet that contained a checklist similar to that used by the SPs that provided benchmarks to guide their self-assessment. On the same worksheet, students were asked to write brief descriptions of their demonstrated strengths and weaknesses, and then identify three goals for improvement and a concrete plan for doing so (see Appendix).

### Group videotape review

After the first and second SP encounter and S-VTR, students reviewed a 2–3 min segment of their interview in class. During this 2 h, faculty facilitated G-VTR, each of the eight students shared their videotape segments. Before showing the clip they were expected to state why they chose this particular segment and to ask the group for focused feedback. The faculty member modeled giving specific feedback on modifiable behaviors and encouraged the students to do so for each other. They did not give explicit feedback on the students SAGS but simply reinforced the benchmarks and modeled identifying behaviorally specific feedback and concrete improvement plans.

### Rating of quality of self-assessment and goal setting

We defined quality of students’ SAGS by the specificity with which the students were able to articulate observations about their interview skills and then delineate a specific and reasonable plan to remedy the perceived deficiencies. A research assistant, blind to interviewing performance, rated the quality of the students’ written SAGS using a three-point scale (poor, fair, good). Table 1 provides criteria (and examples) for rating the quality. A random sample of 31 ‘worksheets’ were re-rated by two faculty members and agreement between the original rating and faculty member A was 83% and faculty member B was 86% and agreement between the faculty raters (faculty members A and B) was 87%, suggesting high inter-rater reliability. For subsequent analyses, poor and fair were combined (given that only 10% of worksheets were rated as ‘poor’) and a dichotomous rating scale of poor/fair versus good was used.

**Table 1:** Rating of the quality of self-assessment and goal setting (SAGS) (n=129)

	Poor	Fair	Good
Initial rating	13 (10%)	70 (54%)	46 (36%)
Criteria for rating	Major elements missing/incomplete Superficial Goals focused on simply needing to improve	Completed Broad, not sufficiently detailed assessment Goals too broad, not actionable Goals and self-assessment not fully aligned	Completed with care Specific, sufficiently detailed assessment Achievable, focused goals Goals closely linked with self-assessment
Examples	*Self-Assessment*: I'm too nervous *Goal Setting*: Don't stress about	*Self-Assessment*: I seem awkward when addressing touchy subjects *Goal Setting*: Make sure focus on being considerate and not judgmental when discussing touchy subjects	*Self-Assessment*: I was no longer making eye contact with the patient when I asked about substance use *Goal Setting*: Think about the patient as a person when I ask about touchy subjects and explore my own feelings about substance use
Dichotomized	Poor 83 (64%)		Good 46 (37%)

### Rating of interviewing skills

In all three exercises, SPs assessed the students’ interviewing skills using a behaviorally anchored, 17-item communication skills checklist that included items in three core communication domains: information gathering, relationship development, and patient education and counseling. Checklist items were rated as not done, partly done, and well done (each with associated behavioral anchors) and an overall interviewing skills score was calculated as the percent of items rated as ‘well done’. Internal consistency of the checklist was strong with Cronbach's alpha ranging from 0.83 to 0.88 across the three SP exercises.

Because the SP exercises were explicitly designed to increase in difficulty over the course of the academic year, percent of items rated well-done for each exercise were transformed into Z-scores (z-scores are expressed as standard deviations above or below the mean) to facilitate the comparison of relative improvement in skills over time while accounting for increases in case-difficulty.

### Statistical analyses

Frequency distributions are reported for the quality of students’ initial SAGS. Simple Chi-square analyses were used to explore changes in the quality of students’ SAGS from Time 1 to Time 2. Analyses of variance (ANOVAs) were used to explore the effects of both time as a repeated measures factor (three time points of assessment) and of quality of SAGS skills (as the between subjects factor, poor vs. good quality), as well as their interaction, on interviewing skills. To explore associations between changes in SAGS skills and subsequent interviewing skills, the between subjects factor included three groups: students whose SAGS skills were poor at both Time 1 and Time 2 (remained poor); students whose SAGS skills were poor at Time 1 but were rated as good at Time 2 (improved); and students whose SAGS skills were rated as good at both Time 1 and Time 2 (remained good). Post hoc analyses with Bonferroni corrections were used to isolate significant differences.

## Results

### Self-assessment and goal setting

#### Quality of initial SAGS

For the 82% (129/157) of students who turned in worksheets after their first SP case, quality of initial SAGS was categorized as follows: 36% (n=46) ‘good’, 54% (n=70) ‘fair’, and 10% (n=13) ‘poor’ (Table 1). For all subsequent analyses, ‘poor’ and ‘fair’ ratings were grouped together because of the small number of worksheets rated as poor, resulting in a dichotomous SAGS quality of either ‘good’ or ‘poor/fair’.

#### Change in quality of SAGS

The percentage of all students with ‘good’ SAGS improved significantly from Time 1 to Time 2 (36%, n=46/129 vs. 61%, 63/104). Table 2 shows that for the 104 students who handed in both SAGS worksheets, 40% (n=41) had poor quality SAGS skills at both Time 1 and Time 2 (remained poor); 27% (n=28) improved from poor quality at Time 1 to good at Time 2 (improved); and 34% (n=35) had good quality SAGS skills at Time 1 and Time 2. No student in this study declined in SAGS quality over the course of the year.

**Table 2:** Change in quality of SAGS from initial to time 2 (week 10) assessment

	Time 2		
		
Initial	‘Poor’ SAGS quality	‘Good’ SAGS quality	Chi-Square
‘Poor’ SAGS quality	41 (40%)	28 (27%)	4.65 p <0.05
‘Good’ SAGS quality	0 (0%)	35 (34%)	

### SAGS and interviewing performance

#### Quality of initial SAGS skills and both concurrent and subsequent interviewing skills

At baseline, the quality of students’ SAGS skills was not associated with their interviewing skills exhibited in the first SP exercise: students judged to have ‘good’ SAGS scored 70% well done on the OSCE (SD=23%), vs. 72% well done (SD=23%) for those with ‘poor’ SAGS (t-test=0.39, p=0.70).

**Fig. 1:**
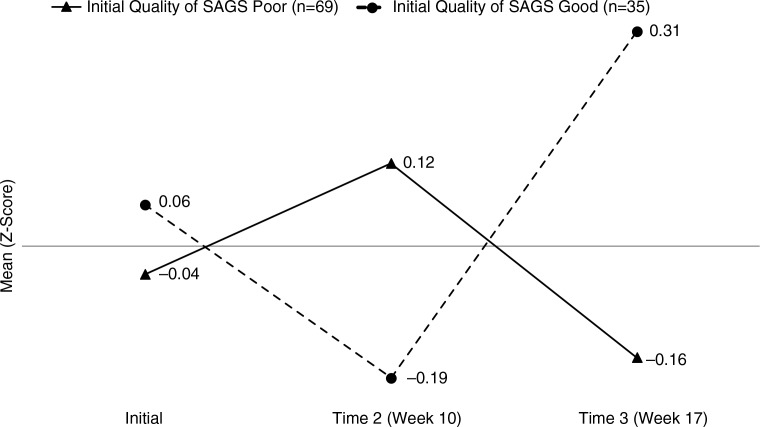
Initial Quality of SAGS and Subsequent Interviewing Skills (n=104). Quality of SAGS x Time Interaction *F=*4.16, p=0.024. Post hoc Bonferroni-corrected pairwise comparisons showed that for those with poor initial quality of SAGS, interviewing scores decreased while for those with good initial quality of SAGS, scores increased.

Initial SAGS skills were associated with a subsequent change in interviewing skills over the three SP exercises (Week 1, Week 10, and Week 17). The main effect of time was significant (F=9.32, p<0.001), with z-scores showing a general increase over time, while the quality of initial SAGS was not (F=1.23, p=0.67). However, the interaction of these two factors was significant (F=4.16, p=0.044) with a pattern suggesting a latency effect (Fig. 1). At Time 1, interviewing skill scores were similar for both those with poor and good quality SAGS skills. At Time 2, students with poor-quality SAGS skills had higher mean interviewing scores than those with good SAGS scores. By Time 3, however, the pattern switched, and students with good baseline SAGS skills had substantially higher interviewing scores than those with poor baseline SAGS skills. These standardized scores show that this statistically significant difference is a moderate sized effect, about 0.5 of a standard deviation (or about 12–15% difference in % items rated as well done on our checklist).

#### Change in quality of SAGs and subsequent interviewing skills

**Fig. 2:**
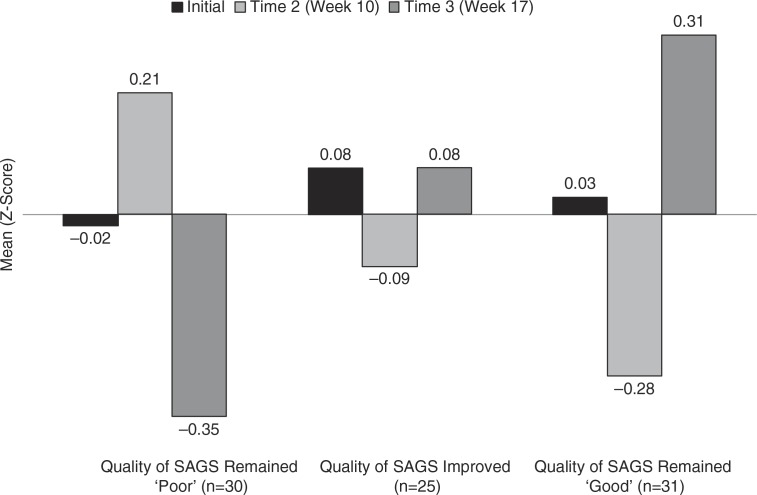
Change in Quality of SAGS and Interviewing Skills Over Time (n=86). ANOVA Change in Quality of SAGS x Time Interaction *F=*2.44, p=0.044. Post hoc Bonferroni-corrected pairwise comparisons showed that the interviewing scores of students whose quality of SAGS rating remained poor significantly decreased from Time 2 to Time 3 while the interviewing scores of those whose SAGS ratings remained good increased from Time 2 to Time 3 (after decreasing from Time 1 to Time 2).

An analysis of variance with time as the repeated measure factor and the change in quality of SAGS skills as the between subjects factor demonstrated a significant interaction effect (F=2.44, p=0.047, n=104). As shown in Fig. 2, the pattern of changes in interviewing skills between Time 2 and Time 3 was dependent on group membership. Students who had consistently poor SAGS skills (Group 1, n=30) improved in their interviewing skills at Time 2 but then decreased substantially at Time 3. Students whose SAGS skills improved (n=31) had interviewing skill scores that hovered around the mean and did not change from Time 2 to Time 3. In addition, those students who had consistently good SAGS skills (n=25) appeared to experience, on average, a decline in their interviewing skills between Time 1 and Time 2 but then a meaningful increase from Time 2 to Time 3.

## Discussion

We describe the evolution of the quality of medical students’ SAGS ability and its relationship to demonstrated interview skills over the course of the first year of medical school. While overall both the quality of students’ SAGS in this domain and interviewing skills improved over time – hopefully a reflection of an effective curriculum – subsets of students based on initial and changed SAGS ability had very different patterns, which may have implications for both curriculum and assessment.

Higher baseline SAGS skills were associated with an initial decline and then a substantial increase in communication scores. This pattern may be attributable to a phenomenon known as expertise reversal in which students with more automatic and unconscious competence *temporarily* do poorly on assessment strategies designed for novices (23). Students with solid interviewing skills who then consciously assess and reflect on those skills may find themselves ‘coming undone’ for the next assessment but are rapidly able to re-integrate those skills with what they learned through self-reflection and goal-setting and demonstrate improved interviewing skills when next assessed. Further exploration into the meta-cognitive, motivational and other characteristics of this group of students is likely to identify a highly self-regulated group, perhaps over 50% of students, who may thrive in a more self-directed curriculum.

Low-quality SAGS’ ability at baseline that remained poor 10-weeks later were risk factors for failing to sustain growth in interviewing skills. While these students’ interviewing skills, on average in comparison to their peers, improved from Time 1 to Time 2, they declined by Time 3. The initial increase could be interpreted as a practice effect, one that could not be sustained by the end of the course. We need to better understand these high-risk students. They are likely a diverse group with respect to engagement in certain aspects of medical training. Given the importance of interviewing skills to patient outcomes in practice this may have significant implications for general clinical competence. Within this group could also be those with poor self-regulation skills in general (24). One might predict that medical students are ‘super students’ given the impressive academic success they need to demonstrate to gain admission. However, researchers using standard measures still identify a surprising, range of self-regulation and self-efficacy among enrolled medical students (25).

Students who went from having poor SAGS skills to good SAGS skills did not demonstrate any change in their interviewing skills. Given that effects for the other groups differed over time in this study, we suspect that the impact of enhanced SAGS skills may not be discernible for some time and therefore that a more longitudinal design might have been able to document a lagged effect. For this group, more time and experience may be needed for their enhanced SAGS skills to lead to improved practice. Further research might benefit from including longer time frames in order to capture these potentially lagged effects.

For students who demonstrate the ability to self-assess and set goals with specificity, instructional strategies like structured self-assessed VTR with group review may be sufficient to ensure competence.

We have found that some students do not acquire adequate SAGS skills through a curriculum where benchmarks are provided and generation of behaviorally specific feedback and concrete goal-setting is modeled and that these students are at risk of not acquiring communication skills on par with their peers. Future investigations should examine whether more explicit feedback on the actual SAGS skills would lead to growth in both areas. Students who do not demonstrate this skill may require more explicit training focused on their SAGS skills in order to stimulate growth in their communication skills. However, it is not yet clear what the natural course of SAGS skills is over time, nor what interventions are likely to be effective for these high-risk students. Self-regulation requires significant motivation and is domain specific – so some students may show the ability to self-monitor and achieve in a domain of great interest to them personally and not in others. A major driver of the call to better integrate foundational knowledge and clinical experiences in medical education is to provide students with increased motivation to learn the foundational knowledge by illustrating it in context of practice (26, 27). Ultimately, medical students in order to honor the tenets of medical professionalism must develop strategies to demonstrate competence in domains where they lack personal motivation (28). SAGS skills may be an important aspect of this professional development challenge.

Our study suffers from many of the limitations associated with evaluating educational innovations: a small sample size, short time frame, and limited generalizability. In addition, our assessment of the quality of SAGS skills is somewhat simplistic and may miss important nuances in pattern of students’ skills, for example, students who are reflective but not skilled in setting goals or students who are able to set focused, actionable goals, but find they are unable to follow through on those goals. Finally, some students failed to complete the worksheets and therefore are not included at all time points and may have affected the results.

## Conclusion

According to the 2008 review by Colthart et al. of the medical education self-assessment literature, ‘there is no solid evidence base within the health professions’ literature which establishes the effectiveness of self-assessment in … influencing learning activity’ (16). Our study begins to build the link between goal setting and development of interviewing skills, demonstrating that better SAGS ability is associated with improvement in performance of interviewing skills in a standardized scenario. Students with poor quality self-assessment skills (40% of our students) did not show this improvement. More work needs to be done to understand the longer-term implications of these findings, and to better understand the constructs being examined, both of which may have implications for tailoring of curriculum, remediation of poorly performing students and medical school admissions criteria.

## Previous presentations

Portions of this work were previously presented at: AAMC Annual Meeting, November 4-9, 2011, Denver, CO, SGIM Annual National Meeting, April 21-May 1, 2010, Minneapolis, MN, the 14th Ottawa Conference on the Assessment of Competence in Medicine and the Healthcare Professions, May 15-20, 2010, Miami FL.

## Appendix

**School of Medicine Student Academic Portfolio: Practice of Medicine Clinical Skills Interview Videotape Review**

**Video Tape Review Assignment**

Review the video using the VTR SELF EVALUATION guidelines below. Assess whether or not you demonstrated the specific skill and then as specifically as you can, assess and explain your performance in the text box provided.

Don't be surprised if the first time you watch the clip you are surprised by your looks, gestures, or the sound of your voice. This is common.

Focus on your interviewing skills using the VTR SELF EVALUATION guidelines.

Be sure to note some things that you think you did well. Don't forget that it is easy to be critical of our own performance. Most interviews, even by the most seasoned clinicians, can be improved in some way!

Share this form with your POM Seminar leader by 5 PM on DATE.

Choose a 2–3 min segment of your interview to share in class. It can be something you thought went well or not so well. Just prepare to tell your colleagues why you chose this part.

VTR SELF-EVALUATION

**Table:**

Communication Skills		Did You Do This?	If did NOT do, explain what happened … specific things you missed or did not so well and why
INFORMATION	Managed the **narrative flow**	☐	
GATHERING	Elicited patient's response using a**ppropriate questions** (non-leading questions, one at a time)	☐	
	**Clarified information** by repeating on an ongoing basis to make sure he/she understood	☐	
	Allowed patient to talk **without interrupting**	☐	
RELATIONSHIP	Communicated **concern** or intention to **help**	☐	
DEVELOPMENT	**Non-verbal behavior** enriched communication with patient (e.g., eye contact, posture)	☐	
	Acknowledged patient's **emotions/feelings** appropriately	☐	
	Was **accepting/non-judgmental** of patient	☐	
	Used **words patient understood** and/or explained jargon	☐	
EDUCATION & COUNSELING	ASK: **Asked questions** to see what patient understood/thought	☐	
	**TELL:** Provided clear explanations/information	☐	
	**ASK:** Asked for **patient to explain** what she understood	☐	

How would you rate your overall communication skills …?

**Table:**

1 Inadequate	2 Marginal	3 Adequate	4 Very Effective
Ineffective communication skills will create clinical problems	Uses some communication skills effectively but others could create clinical problems	Uses most communication skills effectively	Uses all communication skills effectively, minor suggestions would enrich
☐	☐	☐	☐

Overall, what went well?

_____________________________________________

_____________________________________________

_____________________________________________

_____________________________________________

_____________________________________________

List three areas of your interviewing that you would like to improve. Be specific in describing the behaviors you observed that you would like to change. Describe a concrete plan to work on them (in other words, what would you like to do differently)?

**Table:**

	Interviewing Areas In Need of Improvement	Concrete Plan for Improving
**1**		

**2**		

**3**		

What did you learn from seeing yourself that you didn't learn right after the OSCE?

_____________________________________________

_____________________________________________

_____________________________________________

_____________________________________________

Overall, how much did you learn from this self-evaluation?

**Table:**

**1** **Nothing Really** ☐	**2** **Learned A Little Bit** ☐	**3** **Learned Something** ☐	**4** **Learned A Lot** ☐

## References

[1] Gordon MJ (1991). A review of the validity and accuracy of self-assessments in health professions training. Acad Med.

[2] Zimmerman BJ (1990). Self-regulated learning and academic achievement: an overview. Educ Psychol.

[3] Ericsson KA (2004). Deliberate practice and the acquisition and maintenance of expert performance in medicine and related domains. Acad Med.

[4] Emanuel EJ, Fuchs VR (2012). Shortening medical training by 30%. JAMA.

[5] Sibille K, Greene A, Bush J (2011). Preparing physicians for the 21st century: targeting communication skills and the promotion of health behavior change. Ann Behav Sci Med Educ.

[6] Simpson M, Buckman R, Stewart M, Maguire P, Lipkin M, Novack D (1991). Doctor–patient communication: the Toronto consensus statement. BMJ Br Med.

[7] Hampton JR, Harrison MJ, Mitchell JR, Prichard JS, Seymour C (1975). Relative contributions of history-taking, physical examination, and laboratory investigation to diagnosis and management of medical outpatients. Br Med J.

[8] Sandler G (1980). The importance of the history in the medical clinic and the cost of unnecessary tests. Am Heart J.

[9] Kassirer J (1983). Teaching clinical medicine by iterative hypothesis testing. Let's preach what we practice. N Engl J Med.

[10] Yedidia M, Gillespie CC, Kachur E, Schwartz MD, Ockene J, Chepaitis AE (2003). Effect of communications training on medical student performance. JAMA.

[11] Kalet A, Pugnaire M, Cole-Kelly K (2004). Teaching communication in clinical clerkships: models from the macy initiative in health communications. Acad Med.

[12] Maguire P, Pitceathly C (2002). Key communication skills and how to acquire them. BMJ Br Med J.

[13] Kurtz S, Silverman D, Draper J (2005). Teaching and learning communication skills in medicine.

[14] Perera J, Mohamadou G, Kaur S (2010). The use of objective structured self-assessment and peer-feedback (OSSP) for learning communication skills: evaluation using a controlled trial. Adv Health Sci Educ Theory Pract.

[15] Gordon MJ (1992). Self-assessment programs and their implications for health professions training. Acad Med.

[16] Colthart I, Bagnall G, Evans A (2008). The effectiveness of self-assessment on the identification of learner needs, learner activity, and impact on clinical practice: BEME Guide no. 10. Med Teach.

[17] Eva K, Regehr G (2005). Self-assessment in the health professions: a reformulation and research agenda. Acad Med.

[18] Rudy D, Fejfar M (2001). Self-and peer assessment in a first-year communication and interviewing course. Eval Heal Prof.

[19] Chang A, Chou C, Teherani A, Hauer K (2011). Clinical skills-related learning goals of senior medical students after performance feedback. Med Educ.

[20] Stevens DL, King D, Laponis R, Hanley K (2009). Medical students retain pain assessment and management skills long after an experiential curriculum: a controlled study. Pain.

[21] Yedidia MJ, Gillespie CC, Kachur E (2003). Communications training improved student performance: findings from a controlled 3-school experiment. JAMA.

[22] Hochberg MS, Kalet A, Zabar S (2010). Can professionalism be taught? Encouraging evidence. Am J Surg.

[23] Kalyuga S, Ayres P (2003). The expertise reversal effect. Educ Pysch.

[24] Artino AR, Hemmer PA, Durning SJ (2010). Using self-regulated learning theory to understand the beliefs, emotions, and behaviors of struggling medical students. Acad Med.

[25] Song HS, Kalet AL, Plass JL (2011). Assessing medical students’ self-regulation as aptitude in computer-based learning. AHSE.

[26] Cooke M, Irby D, O'Brien B (2010). Educating physicians: a call for reform of medical school and residency.

[27] Koens F, Mann K, Custers E, Ten Cate O (2005). Analysing the concept of context in medical education. Med Educ.

[28] Passi V, Doug M, Peile E, Thistlethwaite J, Johnson N (2010). Developing medical professionalism in future doctors: a systematic review. Int J Med Educ.

